# Neurobehavioral measures of coincidence anticipation timing

**DOI:** 10.1167/jov.23.8.16

**Published:** 2023-08-23

**Authors:** Louise A. Stolz, Sicong Liu, Edem Asamoa, Lawrence Gregory Appelbaum

**Affiliations:** 1 Department of Psychiatry, University of California, San Diego, CA, USA; 2 Annenberg School of Communication, University of Pennsylvania, Philadelphia, PA, USA; 3 Department of Psychiatry and Behavioral Sciences, Duke University School of Medicine, Durham, NC, USA; 4 Department of Psychiatry, University of California, San Diego, CA, USA; 5 Department of Psychiatry and Behavioral Sciences, Duke University School of Medicine, Durham, NC, USA

**Keywords:** coincidence anticipation, timing, EEG, visual-evoked potential

## Abstract

Coincidence anticipation (CA) refers to the ability to coordinate responses to the arrival of a moving object. This study investigates the neurobehavioral processes that underlie CA through the measurement of electroencephalography (EEG) recorded during a CA task on a 17-foot plastic rail with evenly spaced LED lights. Participants responded at the anticipated moment a sequence of successively lit LEDs coincided with a stationary target. Healthy young adult participants (*M*_age_ = 21) performed six blocks with movement at 20, 30, or 40 mph and the direction either inbound or outbound relative to the participant. Behavioral results demonstrated a main effect of speed and an interaction between speed and direction, with outbound motion producing early responses and inbound motion producing delayed responses that increased at greater speeds. EEG demonstrated characteristic P1, N2, and P3-like visual evoked potentials (VEPs). VEP amplitudes revealed a significant direction by channel interaction for the P1, indicative of more medial responses for inbound motion. Significant laterality differences were present in the N2, whereas the P3 component produced significant main effects and interactions of speed and direction. This novel combination of three-dimensional CA with EEG demonstrates systematic brain responses that are tuned for motion speed and sensitive to different egocentric motion patterns thereby shedding new light on the mechanism of human visual-motor control.

## Introduction

The ability to anticipate and respond to movements in the environment is central to many human activities, from everyday actions like shaking hands to feats of athletic marvel, like hitting a homerun. The behavioral processes and neural mechanisms that allow timing control are widely studied areas of psychometric research (Badler & Heinen, 2006; Fleury, Bard , Gagnon, & Teasdale 1992; Gregory, 1986; Müller & Abernethy, 2012) and cognitive neuroscience (Antle & Silver, 2009; Drews, Pacheco, Bastos, & Tani, 2021; Masaki, Sommer, Takasawa, & Yamazaki, 2012). Experimentally, coincidence anticipation (CA) abilities are measured through tasks asking participants to predict the arrival of a moving object at a point in space through a coordinated motor response. While such CA tasks can be used to quantify the behavioral processes and neural mechanisms underlying anticipatory timing of moving objects, no study has yet tested the speed or directional tuning of CA to understand the contribution of early visual cortical processes that underlie these essential human abilities.

Previous studies have used CA tasks to understand the behavioral impacts of timing errors (Gregory, 1986; Ross, Kinney, & Fogt, 2022). In such studies, several measures of response error are typically evaluated. Constant error (CE) calculates the mean inaccuracy and whether the error was early or late (Duncan, Smith, & Lyons, 2013). CE is informative of both the magnitude of an error as well as the direction which can elucidate the mechanism of behavioral responses to various velocities or experimental contexts. Absolute error (AE) is the absolute value of the timing inaccuracy, without consideration of its directionality (i.e., sign), therefore offering a measure of response precision (Rodrigues, Vasconcelos, Barreiros, & Barbosa, 2009). Variable error (VE) refers to consistency, or the deviation, of scores about the subject's own average score, shedding light on the trial-to-trial variability of responses (Akpinar, Devrilmez, & Kirazci, 2012). These three behavioral variables are commonly used in CA studies as they are each informative of the magnitude and bias of errors, allowing researchers to understand cross-sectional and experiential differences across participants (Harrold & Kozar, 2002; Hodges et al., 2021; Rodrigues, Barbosa, Carita, Barreiros, & Vasconcelos, 2012).

Past studies analyzing errors in timing tasks have often used rails or tracks embedded with sequential LEDs, such as the Bassin Anticipatory Timer (Lafayette Instruments) to simulate object motion through space (reviewed in Crocetta et al., 2018). Research using such light rails has investigated topics including the influence of stimulus speed (Duncan et al., 2013; Harrold & Kozar, 2002), the influence of background patterns (Ridenour, 1981), participant fatigue (Duncan et al., 2013; Duncan, Fowler, George, Joyce, & Hankey, 2015), as well as cross-sectional variability because of age (Haywood, 1980), athlete experience (Brady, 1996; Nakamoto & Mori, 2012), handedness (Rodrigues et al., 2012; Stadulis, Eidson, & LeGant, 1990), and gender (Brady, 1996; Sanders, 2011). Studies on movement velocities have generally shown that slower speeds yield early anticipation with responses occurring before the stimulus has reached the target, while faster velocities tend to yield late errors with subjects responding to the stimulus after it has passed the target (Brady, 1996; Ridenour, 1981; Stadulis et al., 1990). In the current study, the same behavioral error measures discussed above will be used to analyze differences between inbound and outbound trajectories at varying speeds and these will be compared to early brain responses measured with electroencephalography (EEG) to test hypotheses about the relation between brain and behavior.

The neural mechanisms of CA have been predominantly studied through measurement of scalp-recorded EEG due to its high temporal resolution. This research has built on studies that have characterized the spatial and temporal distribution of visual evoked potentials (VEP) to motion onset (reviewed in Heinrich, 2007) and the motor potential elicited from manual responses (Hallett, 1994; Shakeel et al., 2015). In visual motion tasks, the evoked response is typically characterized by an early positive component around 100 ms, the so-called *P1 component*, that is thought to reflect change in the visual stimulus that is not specific to movement. This component has been associated with initial sensory and attentional processing of stimuli in the extrastriate and visual cortices (Asanowicz et al., 2020) and has been shown to be specifically modulated by attention which may be relevant to coincidence anticipation. The P1 is followed by an occipital-temporal negative deflection between 150 and 200 ms, the N2 component, that is specific to motion speed, direction, and contrast (Hülsdünker, Ostermann, & Mierau, 2019; Kubová, Kuba, Hubacek, & Vít, 1990; Lorteije, van Wezel, & van der Smagt, 2008; Patel & Azzam, 2005; Vilhelmsen, van der Weel, & van der Meer, 2015). The N2 component originates from the human visual area MT and has been widely associated with visual motion processing and processing of the spatial position of moving objects (Feldman & Freitas, 2019; Hülsdünker et al., 2019; Hülsdünker, Strüder, & Mierau, 2017). Following the N2 is a broad central-parietal positive component that spans from roughly 300 to 500 ms, referred to as the *P3*, which has been associated with attention, decision-making, and reaction to a stimulus, rather than the stimulus itself (Polich, 2003). Changes in P3 amplitude and latency have also been associated with higher level cognitive processes (Asanowicz et al., 2020; Kopp, Steinke, & Visalli, 2020).

At least three previous studies have fused CA tasks with EEG (Koshizawa et al., 2013; Masaki et al., 2012; Nakamoto & Mori, 2012). In one such study by Koshizawa and colleagues (2013), EEG was recorded as participants trained on a CA task. After training, EEG power in the beta frequency range (13-30 Hz) was found to increase, whereas VEP amplitudes over central channels were reduced and occurred earlier, suggesting a change in the balance of sensory processing and response generation. Masaki and colleagues (2012) explored the neural responses to CA by comparing timing errors to the contingent negative variation, a slow-building EEG potential that has been associated with stimulus anticipation and response preparation. They observed that constant timing behavioral errors were higher for high velocity movement and contingent negative variation amplitudes increased with both faster stimulus velocity and increased response duration, suggesting that this pattern reflects increased motor programming efforts for these conditions. Finally, Nakamoto and Mori (Nakamoto & Mori, 2012) analyzed the N2 and P3 components in a CA task in which the velocity of the light-movement changed over time. When comparing baseball experts and novices, they found smaller timing errors, and shorter N2 and P3 peak latencies in experts and suggested that this was due to experience with catching decelerating stimuli from playing baseball.

Although these previous studies have shed light on CA abilities, no study has ever tested how speed and direction of movement manifest in different behavioral and brain responses. Moreover, it is not currently known if speed and directional tuning can be measured with three-dimensional movement (nearly all previous EEG studies were done on two-dimensional monitors) or whether early visual cortical responses are tuned to the direction and speed of movement. In the following study, a CA task with inbound and outbound motion directions was tested with three speeds on a mounted 17-foot (5 m) light rail during concurrent EEG recording. Analyses were conducted to test CE, AE, and VE behavioral reaction time effects and event-related potentials (ERPs), focusing on the P1, N2 and P3 ERP components, described above.

Past studies on the effect of velocity on timing errors have led to disparate results with most studies finding that higher speeds led to greater errors (Duncan et al., 2013; Harrold & Kozar, 2002), whereas others have found the opposite pattern (Masaki et al., 2012). These studies, however, have used considerably different movement types (e.g., linear, radial, and rotational), and therefore in the current study it was hypothesized that behavioral errors would increase with greater speeds because this is the most common finding with linear motion. Furthermore, it was hypothesized that visual evoked potentials would produce higher amplitude for faster speeds but that the two directions of movement would lead to different waveform morphology because of the differing projections of motion over the retina and visual cortex.

This study therefore provides a novel test of human psychophysiology that underlies coincidence anticipation abilities. Developing a clearer understanding of the neural processes underlying direction and speed tuning is relevant to multiple domains such as improving athletic abilities using visuomotor training, as well in neurorehabilitation where patients may be engaging in vision therapy after traumatic brain injury. In particular, individuals with impaired visuomotor capabilities may benefit from direct assessments of their coincidence anticipation abilities and engage in specific therapeutic training to regain normal functioning. However, before such training can be implemented on the field or in a clinic, there is a value in understanding the fundamental neural processes controlling coincidence anticipation, which is the goal of this study.

## Methods

### Participants

Twenty-six participants took part in this study. Two participants were excluded during data collection due to poor compliance with the experimental procedures, resulting in 24 total subjects considered for analyses. Two participants were subsequently excluded during the analyses because of poor behavioral performance that produced differing error distributions among conditions. The final sample, therefore, consisted of 22 participants (*M*_age_ = 21.14, *SD*_age_ = 3.03; female = 15) who self-reported as being either right-handed or ambidextrous, with normal or corrected-to-normal vision. All participants gave informed consent and were compensated $10 per hour. The experimental protocol followed the Declaration of Helsinki and was approved by the Institutional Research Review Board.

### Apparatus and task design

**Figure 1.**
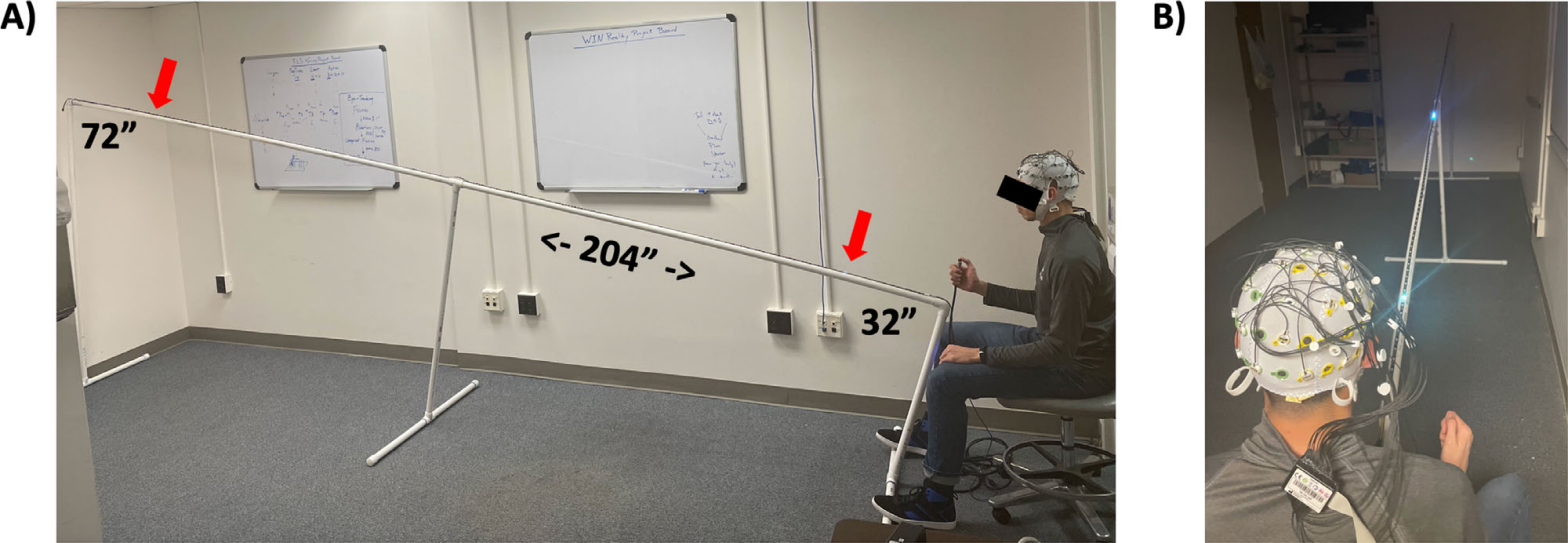
The experimental aparatus shown (**A**) from the side with dimensions in inches, and (**B**) from over the participant's right shoulder with the inbound target LED and a moving LED both lit. Images show the light rail on the PVC stand, 32-channel EEG, and participant pressing the trigger button with their right thumb. Note that the experiment was performed in controlled low-lighting conditions that differ from these images.

This study was performed with a custom combination of devices shown in Figure 1 that consisted of a 17-foot light rail that was mounted on a PVC stand and was connected to a 32-channel mobile EEG system. The light rail (Synchrony; Senaptec LLC, Beaverton, OR, USA) is made of a plastic strip that contains 100 evenly spaced LED lights and is connected to a control box, power source, response button, and digital communication channel to the EEG system. The light rail was supported by a custom PVC stand that stood 72 inches (183 cm) above the floor on the end farthest from the participant and 32 inches (81 cm) above the floor at the end closest to the participant. Through a mobile phone application, the task parameters were preset to define the speed, direction, as well as the start and end point of the LED progression. Stimulus parameters and response data were displayed on the mobile phone application at the end of each block of trials and saved for offline analysis.

EEG signals were recorded with a mobile recording system (LiveAmp 32; Brain Products GmbH, Gilching, Germany). The LiveAmp is comprised of 32 active Ag-AgCl electrodes with preamplifiers in each housing, arranged according to the international 10 to 20 system and referenced relative to channel FCz. Signals were amplified and sent wirelessly, via Bluetooth, to a laptop computer that digitized and recorded the signal at a sampling rate of 500 Hz for subsequent offline analysis. Impedances were set to below 10 kHz at the start of the session to ensure good signal quality.

The experimental design consisted of a two direction (inbound and outbound) by three speed (20, 30, and 40 mph or 32, 48, and 64 km/h) within-participant design. Each of the six conditions were presented as a block of 150 trials and each participant completed a total of six blocks. The order of the blocks was arranged according to a nested counterbalancing, with each of the 24 participants who completed the study receiving one of the six possible orderings of the three speeds, with the order repeated for both directions of motion. Here, speed specified how fast the traveling LEDs were lit in sequence, creating apparent motion, whereas direction indicated the trajectory of the motion relative to the participant's perspective.

During the experiment, participants sat on a chair approximately one foot (30 cm) away from the rail's lower end and faced the light rail end on. The inbound direction corresponded to the light moving from the far end of the rail to the near end of the rail, with a target LED located one foot (ninth LED) from the near end of the rail that remained lit throughout the trial. In the outbound direction, the light moved from the participant towards the far end of the rail, with the target LED located one foot (ninth LED) from the distant end of the rail. It is important to note that while the global motion of the light sequence was toward or away from the participant, because the rail was positioned below the participant's line of sight, inbound movement also projected downward over the visual field, while outbound movement also projected upward in the field.

Participants were instructed to respond with their right thumb to press a response button at the expected moment that the moving lights coincided with the stationary target LED. When the participant pressed the button, the last lit LED remained lit for approximately one second, giving the participant visual feedback of their accuracy relative to the constantly lit target LED. On trials where the participant gave a perfect response and the last lit LED coincided with the target LED. As such, participants could monitor their accuracy on each trial based on the visual feedback provided on the light rail to inform their subsequent estimation of timing. A random interval between 1.8 and 2.1 seconds elapsed between the end of feedback on one trial and the onset of the LED in the next trial.

To minimize EEG recording artifact from eye movement, participants were encouraged to keep their eyes on the static target light when performing the task. Although fixating on the target rather than following the trajectory of motion is not reflective of all natural behaviors, studies comparing pursuit of object motion and steady fixation on CAT have found no difference in perception or a functional relationship between eye movement and CA (Benguigui & Bennett, 2010). Moreover, movement can still be tracked in peripheral vision allowing for similar behavioral impacts (Bennett, Baures, Hecht, & Benguigui, 2010). As such, participants were instructed to fixate on the target to reduce noise in the EEG. Irrespective of the movement direction, the movement of the LED took 544 ms at 20 mph, 363 ms at 30 mph, and 272 ms at 40 mph to reach the target LED.

At the outset of the study, participants were informed of the study procedures and provided informed consent, prior to filling out an online demographic form. Participants were then fitted with an EEG cap and electrodes were prepared for recording. Prior to the start of the recorded data, EEG impedances were tested, and participants practiced several trials to become acclimated to the task. One experimenter facilitated the session by programming the device and implementing the predetermined counterbalanced order of task blocks. Participants then performed the task in a darkened room with the light rail as the only source of illumination. Participants completed five practice trials before performing each task block without interruption and rested for about two minutes between blocks. The full session lasted less than two hours in all cases.

### Measures and statistical analyses

Response time errors and visual evoked potentials are the main measures considered in this experiment. Time errors are defined as the time differential in milliseconds between the participant's response and the moment the moving LED coincided with the stationary target. Negative timing errors indicated early responses. Positive timing errors indicated late responses. Behavioral responses were recorded in the mobile phone application and further verified through the timing of event markers in the EEG recordings.

As is common in the motor control and learning literature (Schmidt, Donald Lee, Winstein, Wulf, & Zelaznik, 2019), coincidence anticipation performance in this study was calculated through three behavioral measures of task performance. Constant Error measures the deviation from the target by averaging the signed errors according to the formula CE = Σ(*x*_i_–*T*)/*n*, where *x*_i_ is the time (ms) elapsed from trial start to response for trial i, *T* is the target time (ms) of perfect performance, and *n* is the number of trials the subject performed. This measure comes with a positive or negative sign, indicating the average bias towards responding early (negative) or late (positive). Absolute Error is a measure of the overall deviation between response time and target time, without consideration of the direction. By calculating the average of the absolute errors according to the formula, AE = Σ|*x*_i_ – *T*|/*n*, where *x*_i_, *T*, and *n* are defined as before, this value describes the accuracy of the average response. Variable Error measures the consistency of the response by calculating the standard deviation of error according to the formula, $\mathrm{VE}=\sqrt{\frac{\sum (x_{i}\;-\;M)2}{n}}$, where *x*_i_ and *n* are defined as before and *M* is the mean response error (i.e., CE).

All EEG measures were preprocessed using the EEGLAB toolbox (Delorme & Makeig, 2004) in Matlab (MathWorks, Natick, MA). To obtain VEPs, all EEG recordings were first bandpass filtered from 0.1 to 30 Hz so that noise coming from environmental electronics (e.g., 60 Hz AC) was attenuated. Stimulus-locked epochs were then generated over the time window from −100 ms before the onset of motion to 600 ms after, and baseline corrected relative to the interval from −100 ms to 0 ms. Zero ms indicates the onset of motion. The resulting epochs underwent artifact rejection with a peak-to-peak threshold of 100 µV imposed. The resulting rejected trials were verified with visual inspection and accounted for less than 10% of total trials.

R (R Core Team, 2019) and SPSS (IBM, Armonk, NY, USA) were used for statistical analyses. Timing errors and VEP response amplitude were tested across speeds and directions through repeated-measure analysis of variance (ANOVA). For behavioral performance, direction by speed ANOVA were performed. Given the novelty of the present task, the VEP analyses were partially informed by visually inspecting the scalp-wide ERP waveforms (see Figure 3). Waveforms contained clear spatial temporal peaks similar to P1, N2, and P3 components reported in other studies testing CA (Nakamoto & Mori, 2012). Therefore VEP analysis focused on occipital, temporal, and central waveforms over the first 600 ms after stimulus onset with the specific channels and latencies selected based on the largest average peak amplitude across participants. Latencies-of-interest were defined as 10 ms before and after the P1 peak, 20 ms before and after the N2 peak, and from 300 to 500 ms for the P3 component. These corresponded to ranges of 82 to 102 ms in the inbound condition and 86 to 106 ms in the outbound condition of the P1, 142–182 ms and 130 to 170 ms for the inbound and outbound N2, respectively. Channels for each component were selected, in part based on the visualized maximal amplitude and spatial distribution. The Cz channel was selected for the P3 component because this component is associated with more central processing, the P7 and P8 channels were used for the N2 component because of its association with the parietal region, and the O1, Oz, and O2 channels were used for the P1 component because of early visual processing associated with the occipital cortex. Speed by channel by direction repeated measures ANOVA were calculated on the mean amplitudes in each of these latency ranges to test the influence of the task design on VEP responses. Greenhouse-Geisser correction on degree-of-freedom was used when Mauchly's test for sphericity reached statistical significance. When post hoc pairwise comparison was needed, the familywise alpha level was controlled using the Holm-Bonferroni method. Statistical significance was set as *p* < 0.05.

## Results

### Behavioral response errors

**Figure 2.**
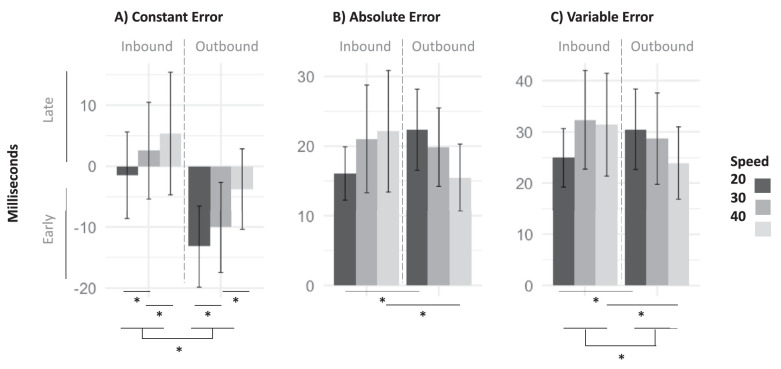
Means and SDs of the experimental conditions for (**A**) constant error, (**B**) absolute error, and (**C**) variable error in the coincidence anticipation task. * = significant effect. Zero is indicative of a perfect timing response (i.e., the participant anticipated the coincidence without any error).

Pairwise comparison among levels of speed across both conditions for CE indicated that 20 mph (*M* = −7.34, *SD* = 5.51) was significantly more negative than 30 mph (*M* = −3.75, *SD* = 6.00), *p* = 0.005, Cohen's *d* = −0.63, which was also significantly more negative than 40 mph (*M* = 0.80, *SD* = 7.22), *p* < 0.001, Cohen's *d* = −0.79. Pairwise comparison between levels of direction showed that outbound movement elicited more negative CE than inbound, *p* < 0.001, Cohen's *d* = 1.67. ANOVA results for CE revealed a significant effect of speed, *F*(2, 42) = 24.44, *p* < 0.001, *η*^2^_p_ = 0.52, suggesting that higher speed is associated with a bias to respond relatively late, and a significant effect of direction, *F*(1, 21) = 61.00, *p* < 0.001, *η*^2^_p_ = 0.74, implying that a bias to respond late is associated with the inbound condition whereas a bias to respond early is associated with the outbound condition. Figure 2A displays these results. The values displayed represent the magnitude and direction of the error, where 0 corresponds to no error.

With regards to AE, pairwise comparison revealed that the interaction was driven by opposing changes in the 20 and 40 mph conditions from inbound to outbound direction. Specifically, the 20-inbound condition (*M* = 16.09, *SD* = 3.83) showed smaller AE than the 20-outbound condition (*M* = 22.36, *SD* = 5.82), *p* < 0.001, Cohen's *d* = −0.99, whereas the 40-inbound condition (*M* = 22.14, *SD* = 8.72) showed larger AE than the 40-outbound condition (*M* = 15.50, *SD* = 4.81), *p* < 0.005, Cohen's *d* = 1.05. ANOVA results for AE showed a speed by direction interaction, *F*(2, 42) = 23.91, *p* < 0.001, *η*^2^_p_ = 0.53. Figure 2B illustrates this interaction. These differences imply that performance was differentially affected by velocity depending on motion direction. Participants performed better for slower velocities in the inbound condition but worse in the outbound condition.

Finally, for VE, pairwise comparison demonstrates that this interaction is driven by opposing changes in the 20 and 40 mph conditions from inbound to outbound direction with 20-inbound (*M* = 24.95, *SD* = 5.71) showing smaller VE than 20-outbound (*M* = 30.52, *SD* = 7.85), *p* = 0.007, Cohen's *d* = −0.91, and 40-inbound condition (*M* = 31.42, *SD* = 10.04) showing larger VE than 40-outbound (*M* = 23.93, *SD* = 7.06), *p* < 0.001, Cohen's *d* = 0.94. ANOVA results for VE revealed an effect of direction, *F*(1, 21) = 6.53, *p* = 0.02, *η*^2^_p_ = 0.24, showing that higher VE occurred in the inbound (*M* = 29.58, *SD* = 6.64) than the outbound (*M* = 27.72, *SD* = 6.69) condition, *p* = 0.02, Cohen's *d* = 0.28. Results also show a speed by direction interaction, *F*(2, 42) = 16.28, *p* < 0.001, *η*^2^_p_ = 0.12. Figure 2C illustrates this interaction.

Collectively, the behavioral results show that higher speeds led to late response bias and participants overestimated the speed of motion in the outbound condition and underestimated it in the inbound condition. AE and VE results are generally consistent in that both became larger when CE was larger in an experimental condition.

### Visual evoked potential results

**Figure 3.**
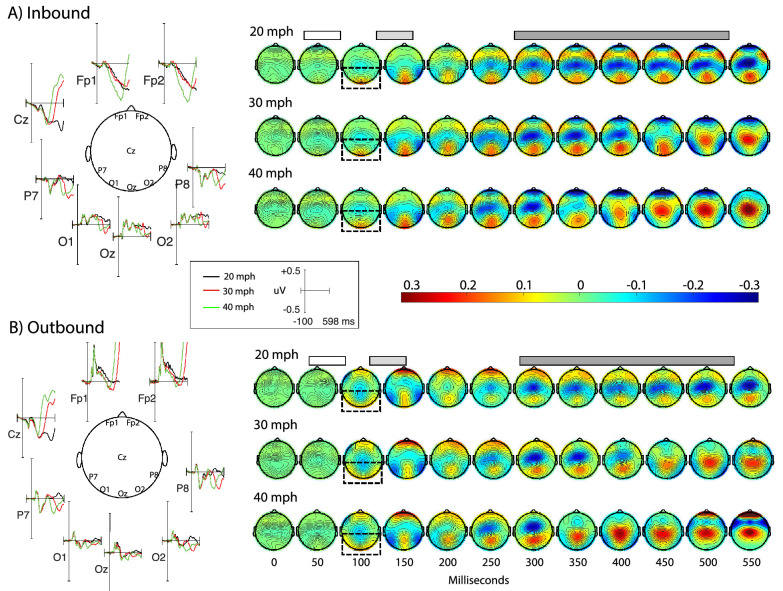
Grand average waveforms and scalp topographies for (**A**) inbound and (**B**) outbound VEPs. On the left, waveforms are shown over frontal, central, and occipital channels-of-interest with trace color differentiating the stimulus speed; 20 mph = black, 30 mph = red, 40 mph = green. On the right are shown grand averaged topographies calculated in 50 ms windows for each speed, with the color bar indicating the mapping between color and amplitude and the gray shading indicating time windows for ERP analyses. Open box = P1, light gray shading = N2, and dark gray shading = P3 components.

Observation of the morphology of the time course and distribution of the inbound (Figure 3A) and outbound (Figure 3B) ERPs show clear visual evoked potentials with peak amplitudes over the first 600 ms that are many times larger than the pre-stimulus baseline activity (See Appendix A and B for illustration of ERPs for all 32 channels). Early P1 evoked responses tended to differ between inbound and outbound trajectories with a more central occipital positivity around 100 ms for inbound and a more bilateral occipital positivity for outbound. At around 120 to 150 ms, the outbound responses contained a pronounced frontal positivity that was not present for inbound stimuli. At latencies after 150 ms, the inbound and outbound conditions elicited more similar distributions with posterior lateral negative and later central positive responses similar to N2 and P3 components reported in other studies (Nakamoto & Mori, 2012). Subsequent analyses focused on the effects of speed, direction, and channel on these components.

**Figure 4.**
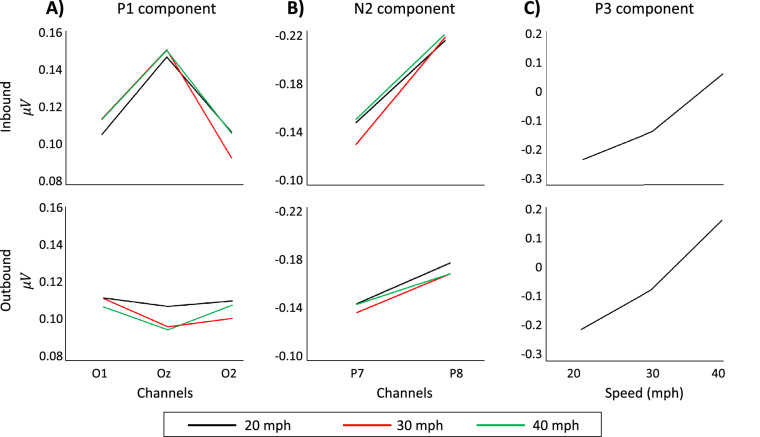
Marginal mean amplitudes for the P1, N2, and P3 components plotted over channels and motion speed for inbound (top) and outbound (bottom) conditions. For panels A and B, black = 20 mph, red = 30 mph, green = 40 mph. (**A**) Mean amplitudes of the P1 component from channels O1, Oz, and O2. (**B**) Mean amplitudes of the N2 component from channels P7 and P8. (**C**) Mean amplitudes of the P3 component from channel Cz.

Three (channels O1, Oz, and O2) by three (speed) by two (direction) ANOVA performed on the mean amplitude of the P1 component revealed a significant direction by channel interaction, *F*(1.69, 35.58) = 4.06, *p* = 0.03, *η*^2^_p_ = 0.16. As shown by the differing occipital distribution of scalp topographies at 100 ms (see dotted boxes) and quantified by the mean amplitudes in Figure 4A, this interaction was driven by a relatively more lateral distribution for the outbound condition and a more medial distribution for the inbound condition. No other main effects or interactions were significant.

Two (channel P7 and P8) by three (speed) by two (direction) ANOVA on the mean N2 amplitude revealed a significant effect of channel, *F*(1, 21) = 5.5, *p* = 0.03, *η*^2^_p_ = 0.21. This difference was driven by larger amplitude N2 responses in left (P7) versus the right (P8) channels, as illustrated in Figure 4B.

Finally, three (speed) by two (direction) ANOVA performed on the mean amplitude of the P3 at channel Cz revealed a significant main effect of speed, *F*(1.41, 29.43) = 113.02, *p* < 0.001, *η*^2^_p_ = .84, a significant main effect of direction, *F*(1, 21) = 18.24, *p* < 0.001, *η*^2^_p_ = .47, and a significant speed by direction interaction, *F*(1.71, 35.85) = 4.70, *p* = 0.02, *η*^2^_p_ = 0.18. As illustrated in Figure 4C, these effects are driven by higher amplitudes at faster speeds, greater amplitude for outbound stimuli, and an interaction between speed and direction in which greater amplitudes are observed at higher speeds for outbound stimuli. Additional analyses correlating the mean amplitude of the P1, N2 and P3 components, averaged over channels, and the constant error, averaged across speeds, were not significant for either inbound or outbound directions. A two-tailed, unpaired *t*-statistic was derived and tested for significance for all six correlations. None were less than 0.4.

## Discussion

This study analyzed behavioral and neural data from a coincidence anticipation task performed on a suspended light rail, under differing speeds and inbound and outbound motion directions. Behavioral results indicate a significant effect of speed and direction on timing errors where outbound movement led to early responses and inbound movement led to later timing errors. Additionally, higher speeds for both directions led to greater timing errors. With regards to EEG responses, different CA conditions led to different ERP morphologies with a significant direction by channel interaction for the P1, laterality differences for the N2, and main effects of speed and direction, and their interaction for the P3. These results demonstrate preliminary evidence that behavioral and neural speed tuning functions can be derived from a coincidence anticipation task that mimics real physical motion in three dimensions. In the following sections we describe first the behavioral findings, then the VEP findings, and then the limitations and future direction for this research.

### Behavioral findings

Behavioral results confirmed findings from prior studies that have found that faster moving objects lead to later responses whereas slower objects lead to an early response bias (Brady, 1996; Ridenour, 1981; Stadulis et al., 1990). The effect of velocity on timing errors is varied in the literature, with some groups finding that higher speeds lead to greater errors and vice versa. In their CA studies, Harrold et al. (Harrold & Kozar, 2002) and Stadulis et al. (1990) observed that slower velocities led to greater errors and an early response compared to faster speeds. They hypothesized that slower velocities led to greater errors because participants change their expectations for and responses to slow velocities, and this delay in neural processing results in larger temporal errors (Harrold & Kozar, 2002; Stadulis et al., 1990). One possible explanation for this finding is that individuals develop an internal model of object motion based on physical principles that are updated through experience, and particularly the angular velocity of the movement as it relates to the current study. Interestingly, although previous studies have found late response bias at higher speeds and early bias at slower speeds, the opposite has been identified in the outbound direction in this study. Several possible explanations exist for these differences.

Angular velocity of an object's motion plays a key role in the calculation of object motion (Kaiser & Calderone, 1991) and may explain the differences observed in constant error performance for the two trajectories. In the outbound condition, earlier responses may stem from the more substantial change in angular velocity earlier in the movement path when the stimuli are closer to the eyes (i.e., the light travels fastest angularly to participants’ eyes at the beginning for each speed condition because of being at a shorter distance/radius to eyes). This may lead to the perception of a faster velocity and earlier response bias. Namely, participant's internal model of estimating the light's movement starts high, and although they may perceive the slowdown of the light and thus downwardly adjust the speed parameter in their internal model, their updating of speed parameter would be slower than necessary and result in generally using a higher-than-real speed parameter. They thus anticipate the light is going to hit the target sooner than reality, showing the early-response tendency in general in the outbound condition. Conversely for the inbound condition, the object appears to be changing angular velocity less over the early stages of movement leading to a delayed response error. Another possibility is that because the light rail was placed below the participant, the inbound motion also projected downward through the visual field, whereas outbound projected upward. This correspondence may also have contributed to differences for the two trajectories. Future studies that systematically manipulate the acceleration and deceleration of movement, or vary the position of the rail, may be able to further substantiate and elaborate these inferences.

Another important element of this design that may have led to differences for inbound and outbound motion is interpretation of the feedback cue. At the end of each trial, the last moving LED and the target LED remained lit, offering feedback about the accuracy of responses. It is possible that this feedback may be interpreted differently for the two movement directions because of their proximity to the observer and as seen in Jensen, Picado, and Morenz (1981), this perspective may have led to differences in the ability to adjust responses for the two directions. Future studies may wish to manipulate the feedback signal, or test performance in the absence of feedback to better understand its role in CAT.

### Visual evoked potential findings

Few previous studies (Koshizawa et al., 2013; Masaki et al., 2012; Nakamoto & Mori, 2012) have attempted to calculate evoked potentials during CA, and therefore this study offers an early proof-of-principle that such measures can be reliably collected. The VEP waveforms here showed a sequence of occipital, central, and frontal components many times larger than the pre-stimulus baseline noise. Interestingly, while the overall morphology of the evoked responses was similar for the two trajectories, an initial early frontal positive response was present in the outbound condition around 150 ms that was not present for inbound stimuli, possibly indicating rapid eye movements or blinks induced by the motion onset that differ for the two movement trajectories.

Results from ANOVA on the P1 mean amplitudes revealed a significant direction by channel interaction indicative of a more medial response for inbound motion and more lateral occipital response for outbound motion. Given the early latency at around 80 to 100 ms, this response may reflect sensory processing in the visual cortex that differs in scalp distribution because of differences in the area of retinotopic activation for motion for the two directions (Wandell & Winawer, 2011). Past studies have identified similar retinotopic dependency of the visual evoked potential, as associated source generators, for components in the time range from 70 to 110 ms post stimulus onset (Clark, Fan, & Hillyard, 1994; Di Russo, Martínez, Sereno, Pitzalis, & Hillyard, 2002).

Analysis of the N2 demonstrated laterality differences with more negative responses in the left, than the right hemisphere, consistent with past reports of asymmetric N2 profiles (Hülsdünker, Ostermann, & Mierau, 2020). Unlike past studies (Lorteije et al., 2008; Vilhelmsen et al., 2015), the current findings did not observe differences in the N2 as a function of stimulus speed, possibly due to the relatively low speed saturation of the N2 component (Müller, Göpfert, Breuer, & Greenlee, 1998) and the fast motion trajectories used for all conditions in this task as compared to previous studies that have found N2 amplitude tuning over slower speeds. Moreover, there were surprisingly no differences in the N2 component between directions either. One possible explanation is that participants were instructed to fixate on the target rather than track the movement so neural processes required to process the different conditions may be negated as eye movement was fixed for all conditions.

Finally, a robust late central-medial positivity was observed in the time range from about 300 to 500 ms, reminiscent of the P3 component reported by Nakamoto and Mori (Nakamoto & Mori, 2012). ANOVA performed on the mean amplitude of this P3-like component produced a significant main effect of speed with more positive amplitudes at higher speeds. This finding validated one of the hypothesized effects of this study and may reflect greater attentional allocation to faster speeds. In addition, ANOVA confirmed a significant interaction between speed and direction, with greater amplitudes at higher speeds for outbound motion. Additional analyses evaluating the correlation between component amplitudes and timing errors did not reveal significant relationships, possibly because of the limited number of participants in this study.

### Limitations and future directions

The current study sought to measure behavior and brain responses during CA over different speeds on movement directions using a commercially available timing device, the Senaptec Synchrony. Given the programming and form factor of the device, certain parameters were pre-determined, such as the intertrial interval, whereas others such as the instructions to fixate on the target were chosen to formalize the design. Future studies may improve on the current design in several ways. First, by extending the timing between successive trials so that there is more than 2.1 seconds to prepare for upcoming trials, participants be better able to prepare and brain responses may have more time to return to baseline. By testing CAT sequences that have movement speeds randomized, rather than blocked it may be possible to better understand the role of expectations on CAT precision as well as the underlying mechanism of action, as has been done previously with EEG during Stroop color-word naming tasks (Appelbaum, Boehler, Won, Davis, & Woldorff, 2012). Furthermore, future studies may benefit from including response-locked analyses to better understand the cascade of brain activation leading to response preparation/inhibition and error prediction, as is frequently done in motor control tasks (Berchicci, Spinelli, & Di Russo, 2016; Sinai, Goffaux, & Phillips, 2007).

Another important point to consider is the instructions and strategy of the participants for maintaining fixation on the CA target during the task. In this case, we chose to instruct the participant to fixate on the target light for several reasons. First, we wished to minimize eye artifacts that would be created by a rapid saccade to track the movement. Secondly, maintaining fixation on the target was thought to be similar to a predictive saccade in which an individual chooses a stable location to saccade in order to place the eyes ahead of movement when movement speeds are too rapid for smooth pursuit, as is the case in baseball (Liu, Edmunds, Burris, & Appelbaum, 2020), as well as in this task. In contrast to batting sports such as softball, baseball, and cricket, this CA task on a light rail does not present a pitcher who goes through a wind-up for the participant to glean “advanced cues” (such as their grip on the ball or their body kinematics), and therefore positioning the eyes near the target might be the most informative location for accurate performance. Finally, it was viewed that instructing the participant to maintain a constant fixation offered the most concrete instructions that would reduce variability and produce a more controlled study to assess behavior and brain activity. Despite these points, as past research demonstrating no difference in performance for tasks that compare pursuit of object motion and steady fixation on CAT (Benguigui & Bennett, 2010), future studies may wish to specifically test these conditions with the use of both EEG and eye tracking to better reveal participant strategies and their underlying mechanisms.

## Conclusions

To conclude, this study provides a preliminary proof-of-concept that speed and direction tuning can be measures behaviorally through behavioral errors and visual evoked potentials from a three-dimensional coincidence. Behaviorally, this study confirmed previous speed tuning findings, while adding an element of direction tuning to better demonstrate biases in response error. Moreover, the addition of VEP offers novel evidence of early visual cortical responses to motion in a realistic simulation of coincidence anticipation.

## Figures and Tables

**Figure A1.**
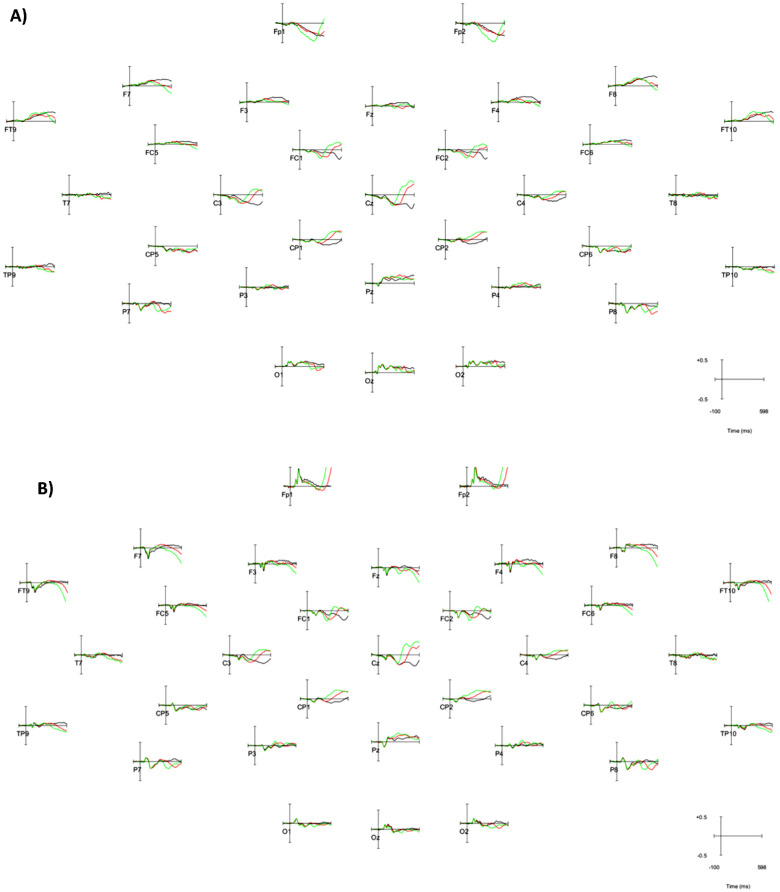
Appendix A and B: Inbound (A) and outbound (B) ERPs of all 32 channels.
